# Vascular Niche in Lung Alveolar Development, Homeostasis, and Regeneration

**DOI:** 10.3389/fbioe.2019.00318

**Published:** 2019-11-12

**Authors:** Akiko Mammoto, Tadanori Mammoto

**Affiliations:** ^1^Department of Pediatrics, Medical College of Wisconsin, Milwaukee, WI, United States; ^2^Department of Cell Biology, Neurobiology and Anatomy, Medical College of Wisconsin, Milwaukee, WI, United States

**Keywords:** endothelial cell (EC), vascular niche, alveoli, lung development, repair

## Abstract

Endothelial cells (ECs) constitute small capillary blood vessels and contribute to delivery of nutrients, oxygen and cellular components to the local tissues, as well as to removal of carbon dioxide and waste products from the tissues. Besides these fundamental functions, accumulating evidence indicates that capillary ECs form the vascular niche. In the vascular niche, ECs reciprocally crosstalk with resident cells such as epithelial cells, mesenchymal cells, and immune cells to regulate development, homeostasis, and regeneration in various organs. Capillary ECs supply paracrine factors, called angiocrine factors, to the adjacent cells in the niche and orchestrate these processes. Although the vascular niche is anatomically and functionally well-characterized in several organs such as bone marrow and neurons, the effects of endothelial signals on other resident cells and anatomy of the vascular niche in the lung have not been well-explored. This review discusses the role of alveolar capillary ECs in the vascular niche during development, homeostasis and regeneration.

## Introduction

Oxygen is an indispensable element in the human body because it is required to generate energy as a form of ATP through a process of cellular respiration in which carbon dioxide is generated as a by-product in mitochondria. Oxygenation of circulating blood and removal of carbon dioxide take place through the alveolar membrane, a thin (0.6–2 mm) and sophisticated barrier structure that is composed of alveolar epithelial cells, intermediate basement membrane (BM) and capillary endothelial cells (ECs) in the lung. Therefore, the lung is one of the most vital organs in the human body. An adult human breathes ~5–8 liters of air per minute (about 10,000 liters per day) to exchange oxygen and carbon dioxide through the alveolar membrane. These membranes are continuously exposed to various toxins or pathogens in the air, and consequently the alveolar membrane is vulnerable to the outer gaseous environment. Although adult lung is thought to be relatively quiescent, specific repair programs are constantly employed to maintain or repair the alveolar membrane in the alveoli. These repair programs are operated by well-organized cooperation of tissue resident cells in the alveoli.

When these repair programs are hampered by aging or an impaired self-defense system, end-stage lung diseases such as chronic obstructive pulmonary disease (COPD), pulmonary fibrosis, and cystic fibrosis ensue. Lung transplantation is the only available therapy for patients with end-stage lung diseases; however, the shortage of donor organs, aggressive surgical procedure, huge economic cost, necessity of life-long immunosuppressive treatment and limited long-term graft engraftment present significant challenges (Mahida et al., 2012; Konigshoff et al., 2013). Therefore, it is critically important to understand the innate alveolar repair programs in order to prevent or treat end-stage lung diseases.

In addition to alveolar epithelial cells, alveolar capillary ECs constitute another integral part of the alveoli. Although the mechanisms by which alveolar epithelial progenitor/stem cells regulate alveolar development, homeostasis, and regeneration have been well-explored (Rawlins and Hogan, 2006; Hogan et al., 2014; Kotton and Morrisey, 2014), less is known about the contribution of alveolar capillary ECs to these processes.

Vascular ECs constitute the inner lining of capillary blood vessels and operate fundamental functions such as delivery of oxygen, nutrients and cellular components, while removing carbon dioxide and cellular waste. ECs are also involved in angiogenesis, microcirculation, coagulation, and inflammation in the local tissues (Cines et al., 1998). Besides these functions, capillary ECs form specific microenvironment, named vascular niche, in which capillary ECs reciprocally interact with other resident cells to regulate development, homeostasis and regeneration in various organs (Lazarus et al., 2011; Ramasamy et al., 2015; Rafii et al., 2016a). Although microanatomy and functionalities of the vascular niche are relatively well-characterized in bone marrow (Kopp et al., 2005; Morrison and Scadden, 2014) and neurons (Ottone et al., 2014; Licht and Keshet, 2015; Karakatsani et al., 2019), the vascular niche in the lung has not been well-characterized. In this review, we discuss the reciprocal interactions between capillary ECs and other resident cells in the alveoli during development, homeostasis, and regeneration. Characterizing the role of ECs in the niche may not only help to understand the mechanisms of lung biology, but also lead to the development of efficient therapeutic strategies for various lung diseases. Moreover, a greater understanding of the niche may identify ways to *ex vivo* engineer functional adult lung tissue that could be implanted into patients with end-stage lung disease. Since the program utilized during organ development is partly utilized in the process of homeostasis and regeneration, we start by discussing the development of alveolar capillaries in the lung. We also discuss the importance of capillary ECs in the vascular niche for homeostasis and regeneration of adult lung.

## Lung Development

Lung development has been studied for many years with an emphasis on elucidating the mechanisms that control differentiation and morphogenesis of airway epithelial cells. Although spatio-temporal interactions between alveolar ECs and other resident cells (e.g., airway epithelial cells, mesenchymal cells, immune cells) play an important role in alveolar development, the role of alveolar ECs in this process has not been well-reviewed.

## Embryonic Stage (E9.5-12 in Mouse, 3–7 Weeks in Human)

**Figure 1:**
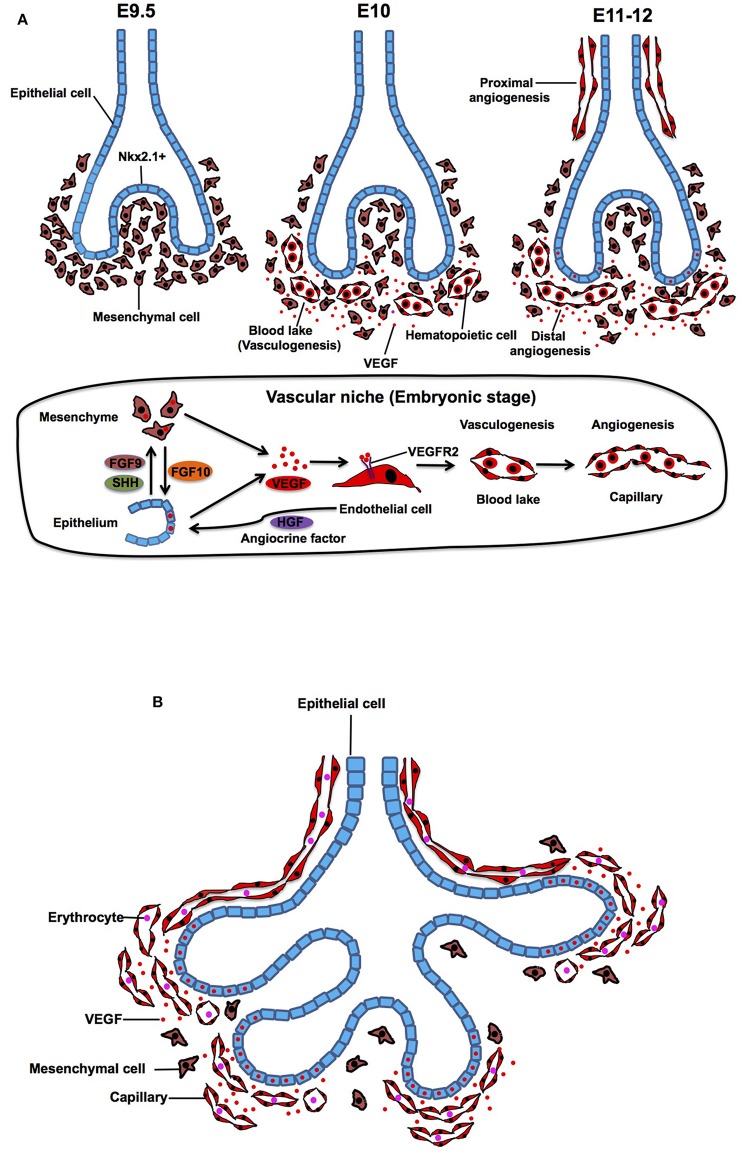
Embryonic and pesudoglandular stage. **(A)** During the embryonic stage, NKX2.1+ epithelial buds grow and protrude into the mesenchyme (E9.5). Density of the mesenchyme becomes sparse and mesenchymal cells start expressing VEGF and stimulate formation of blood lake (vasculogenesis), which is consisted of outer thin endothelial cells (ECs) and inner hematopoietic cells (E10). Blood lakes proliferate, migrate and coalesce into a primitive capillary plexus around the buds (angiogenesis) (E11-12). In the vascular niche, expression of VEGF, which stimulates alveolar capillary network around the buds, is controlled by epithelium-derived morphogens such as FGF9 and SHH. Mesenchyme-derived FGF10 also triggers the production of VEGF in the epithelium. Capillary EC-derived HGF controls lineage commitment in the airway epithelium. **(B)** During the pesudoglandular stage, repeated epithelial branching starts and proximal artery and vein start communicating with the capillaries around the buds. Mesenchyme becomes sparse and the main source of VEGF shifts from mesenchyme to the epithelium, which results in attraction of capillary ECs toward the epithelium.

At embryonic day (E) 9.5 in mouse, primary lung buds are derived from anterior foregut endoderm, which is clearly marked by a homeodomain transcription factor Nkx2.1 (Lazzaro et al., 1991; Kimura et al., 1996). Subsequently, the bud grows and splits into prospective left and right lobes that protrude into the mesenchyme. At E10, density of the mesenchyme around the buds becomes sparse and mesenchymal cells start expressing abundant vascular endothelial growth factor (VEGF) (Shifren et al., 1994; Gebb and Shannon, 2000; Greenberg et al., 2002; White et al., 2007), which is a ligand for VEGF receptor 2 (VEGFR2) on ECs and plays important roles in vasculogenesis and angiogenesis (Chung and Ferrara, 2011; Patel-Hett and D'Amore, 2011; Karaman et al., 2018; Apte et al., 2019). Expression of VEGF, which stimulates alveolar capillary network around the buds, is controlled by epithelial-derived morphogens such as FGF9 and SHH (White et al., 2007). In response to VEGF, hemangioblasts, a subpopulation of mesenchymal cells, form blood lakes in the mesenchyme (vasculogenesis) (deMello et al., 1997; Drake, 2003; Patan, 2004). Morphologically, the blood lakes are formed by outer VEGFR2-positive thin ECs and inner hematopoietic cells (Yamaguchi et al., 1993; deMello et al., 1997; Gebb and Shannon, 2000) (Figure 1A). These blood lakes are closely positioned to the epithelium and predominantly located in the mesenchyme around the distal tips of the epithelial buds (Gebb and Shannon, 2000), suggesting that airway epithelium, mesenchymal cells, and ECs cooperatively interact to form the primitive niche and regulate early epithelial and vascular morphogenesis at this stage. Besides well-known mesoderm-derived EC lineage, lung-specific EC lineage may be derived from Nkx2.1-positive endoderm (Bostrom et al., 2018), suggesting that heterogeneity of ECs already exists at the early developmental stage of the lung.

At E11 in the mouse lung, the proximal vessels now can be clearly identified as vascular tubes that run alongside the trachea, and blood vessels sprout from these larger blood vessels (proximal angiogenesis) (Gebb and Shannon, 2000). Density of the blood lakes increases in the mesenchyme (deMello et al., 1997), and ECs in the blood lake proliferate, migrate and coalesce into a primitive capillary plexus (distal angiogenesis) around the epithelial buds at E12 of mouse lung development (Figure 1A) (Moore and Metcalf, 1970; Pardanaud et al., 1987; deMello et al., 1997; Gebb and Shannon, 2000; Drake, 2003; Parera et al., 2005). Branching of the lung buds and lineage specifications of the airway epithelium are mainly orchestrated by interactions between mesenchyme-derived FGF10 and its counter receptor, FGFR2b on epithelium (Bellusci et al., 1997; Park et al., 1998). Importantly, mesenchyme-derived FGF10 also stimulates epithelial mTORC1/Spry2 signaling, and this signaling triggers the production of VEGF in the epithelium (Scott et al., 2010), which in turn stimulates migration and proliferation of ECs in the mesenchyme around the buds. Conditional VEGF knockdown in distal and proximal airway epithelial cells exhibits disrupted distal vascular network and arrested epithelial branching (Akeson et al., 2003, 2005). Recirpocal interaction among epithelium, mesenchyme and endothelium modulates lung morphogenesis through VEGF signaling in the niche.

While mesenchymal cells play key roles in lineage specification (e.g., Nkx2-1 expression) and epithelial morphogenesis (Shannon and Hyatt, 2004; McCulley et al., 2015), capillary ECs adjacent to the distal airway epithelium (Parera et al., 2005) are also involved in lung-specific lineage commitment in the airway epithelium (e.g., Nkx2.1 expression) by modulating EC-derived morphogens such as HGF (Havrilak et al., 2017; Yao et al., 2017). Capillary ECs also control specification of respiratory progenitors and early budding morphogenesis through non-specific capillary functions (e.g., oxygen and nutrients supply) (Havrilak et al., 2017). These findings suggest that capillary ECs form the vascular niche with other resident cells (epithelial and mesenchymal cells) and play important roles in the primitive stage of the lung development.

## Pseudoglandular Stage (E12-16.5 in Mouse, 5–17 Weeks in Human)

The pseudoglandular stage is characterized by repeated dichotomous branching of the original lung buds. During the pseudoglandular stage, the airway tubes are lined by a high columnar epithelium at the proximal region, and the height of the cells decreases continuously toward the periphery to reach a cuboidal shape in the terminal branches. During this stage, the proximal vascular system, which arises from the sixth aortic arch, develops parallel to the bronchial tree so that all of the pre-alveolar capillary and vein are formed in the characteristic pattern by the end of this stage. When active epithelial branching morphogenesis starts, the mesenchymal cells become apoptotic and density of the mesenchyme between epithelium and ECs gets sparse (deMello et al., 1997). At this stage, the main source of VEGF starts shifting from mesenchyme to epithelium (Yang et al., 2016). Consequently, the epithelium attracts ECs and the buds are completely covered by a polygonal capillary plexus that is formed by Tie2-, PECAM-1, and VEGFR-positive ECs (Parera et al., 2005) (Figure 1B). The capillary plexus contains a lumen filled with erythrocytes, indicating a direct connection between capillaries and embryonic circulation at this stage (Parera et al., 2005).

FGF9 is expressed in epithelium during this stage to regulate FGF10 expression in mesenchyme (White et al., 2007), which also dictates morphogenesis and differentiation of distal airway epithelium (Volckaert et al., 2013). Fgf9^−/−^ mouse lungs show a significant reduction in the distal capillary network density overlying airway epithelium (Greenberg et al., 2002; White et al., 2007), suggesting that epithelium- and/or mesenchyme-derived morphogens regulate capillary morphogenesis. On the contrary, ablation of capillary blood vessels disrupts epithelial morphology (Lazarus et al., 2011), and alveolar capillaries regulate epithelial morphogenesis by generating HGF (Yamamoto et al., 2007). These findings suggest that interdependent crosstalk between epithelium, capillary ECs and mesenchyme in the niche regulates epithelial and capillary morphogenesis at this stage. It is demonstrated that lung epithelial branching morphogenesis does not require capillary ECs in the explant culture (Havrilak and Shannon, 2015). However, physiological mechanical forces such as blood flow in the capillaries and distention of epithelial airways caused by intrauterine-breathing movement, which play crucial roles in epithelial branching morphogenesis are missing in the conventional lung explant culture. Using more sophisticated *ex vivo* culture systems that recapitulate physiological mechanical forces (e.g., miniature bioreactor) (Petersen et al., 2011; Raredon et al., 2016) may further clarify physiological interaction between capillary ECs and epithelial cells in the niche.

## Canalicular Stage (E16.5–17.5 Days in Mouse, 16–26 Weeks in Human)

**Figure 2:**
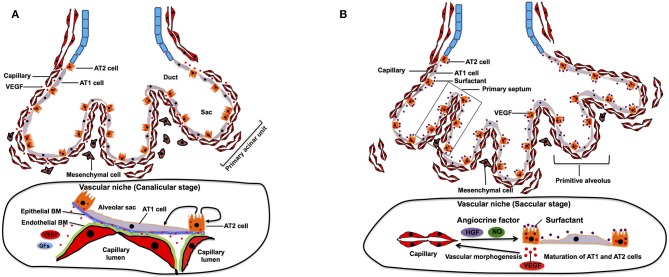
Canalicular and saccular stage. **(A)** During canalicular stage, distal airway is further expanded and vascularized by capillaries. Distal multipotent epithelial progenitor cells differentiate into AT1 and AT2 at the alveolar wall. The source of VEGF is completely shifted from mesenchyme to epithelium, which facilitates closer interaction between epithelium and endothelium. This close interaction also contributes to differentiation of the epithelial progenitor cells into AT1 and AT2 cells. Endothelial and epithelial basement membrane (BM) fuse to form layered BM. BM-bound VEGF and other growth factors (GFs) modulate self-renewal and differentiation of AT2 cells. **(B)** During saccular stage, primitive alveoli are formed, in which alveolar epithelium and capillary endothelium form the interface for gas-exchange by sharing their basement membrane. This process includes further thinning of AT1 cells. Thick primary septa, which include double-layered capillaries, are formed between the walls of primitive alveoli. EC-derived angiocrine factors such as HGF and NO stimulate alveolar epithelial cell maturation and morphogenesis.

After completion of the branching morphogenesis in pseudoglandular stage, the canalicular stage begins in which the respiratory tree is further expanded in diameter and length and characterized by further vascularization along the airway. The pulmonary acinar units, which contain alveolar ducts and alveolar sacs are formed during this period. While capillaries in the mesenchyme move toward the epithelium by the gradient of epithelium-derived VEGF, distal multipotent epithelial progenitor cells (Id2+, Sox9+, Pdpn+, Sftpc+) differentiate into alveolar type (AT) 1 and AT2 cells (Makanya et al., 2001, 2007). AT1 cells form a thin sheet-like structure covering most of the inner surface area of the terminal airway, whereas AT2 cells are interposed between the sheets of AT1 cells (Schittny, 2017). At this stage, VEGF production is completely shifted from mesenchyme to epithelium (mainly AT2 cells) (Kaner and Crystal, 2001; Pham et al., 2002). The epithelium further attracts capillaries (Yang et al., 2016), and their close interactions induce thinning of the epithelium and differentiation of epithelial progenitor cells into AT1 and AT2 cells (Figure 2A). Inhibition of epithelial-derived VEGF results in abnormal vascular and epithelial morphogenesis in allograft experiment (Zhao et al., 2005), suggesting that reciprocal interactions between epithelial cells and ECs through VEGF-VEGFR signaling play crucial roles in the formation of primitive acinar units. At their contact surface, AT1 cells and ECs form a sheet like structure and are separated by a common layered basement membrane (BM), which is formed by fusion of endothelial and epithelial-derived BM (Schittny, 2017). Extracellular matrix (ECM) acts not only as a physical scaffold, but also as a source of growth factors and biochemical signaling (Hynes, 2009; Ishihara et al., 2018). Therefore, ECM modulates self-renewal and differentiation of stem cells and represents an essential part of the stem cell niche (Nikolova et al., 2007; Brizzi et al., 2012; Ahmed and Ffrench-Constant, 2016). Given that ECM produced by pulmonary ECs can induce proliferation and differentiation of AT2 cells *in vitro* (Adamson and Young, 1996), EC-derived ECM may form an integral part of the vascular niche and play important roles in development of distal airways at this stage.

## Saccular Stage (E17.5 to P5 in Mouse, 26–36 Weeks in Human)

The saccular stage is characterized by alveolar sac formation (primitive alveoli), surfactant production, and further expansion of the capillary networks around the airway (Compernolle et al., 2002). This stage is a pre-requisite for establishment of gas-exchange units in the alveoli. Tremendous expansion of the prospective airway surface during this stage is accompanied by a significant increase in capillary growth and endothelial heterogeneity (Guo et al., 2019). Expression of epithelium-derived VEGF further increases (Bhatt et al., 2000), and the capillary surface area significantly expand at this stage (Burri, 1984). Through reduction of the mesenchyme due to apoptosis of mesenchymal cells (Kresch et al., 1998) and a VEGF gradient, the distance between the capillary endothelium and the alveolar epithelial surface diminishes to prepare for effective gas exchange. The lateral walls of the distal sac approach each other to form the thick primary septa, and dense capillary networks that cover each distal airway sac invaginate into the septa to form double capillary layers (Figure 2B). Presence of capillaries seems to be required for the initiation of primary septation, because lung ECs express HGF, and selective deletion of the HGF receptor gene in respiratory epithelium leads to malformation of the septa (Yamamoto et al., 2007). Another major change during this period is maturation of AT2 cells, which accelerates secretion of surfactant protein to reduce alveolar surface tension for post-natal air breathing (Young et al., 1991). Endothelial-derived nitric oxide (NO) not only induces maturation of AT1 cells, but also stimulates AKT/eNOS signaling in ECs and upregulates the production of surfactant proteins in AT2 cells (Coulombe et al., 2019). These findings suggest that the EC-derived angiocrine factors (e.g., HGF, NO) play crucial roles in the formation of primitive alveolar septa at this stage.

Combinations of various pre-natal conditions (e.g., preeclampsia, chorioamnionitis), subsequent pre-mature birth at this stage and post-natal challenges (e.g., mechanical ventilation, oxygen therapy) result in developmental lung disorders including bronchopulmonary dysplasia (BPD) in preterm neonates (Burri, 1984; Balany and Bhandari, 2015; Schittny, 2017). Lungs of the BPD patients exhibit decreased alveolar capillary density and impaired septation (Meller and Bhandari, 2012; Stark et al., 2018). Epithelial-derived VEGF and endothelial VEGFR2 are significantly downregulated in the lungs of BPD patients (Bhatt et al., 2001), and inhibition of angiogenesis by blocking VEGF receptor at this stage phenocopies BPD pathology (Jakkula et al., 2000; Le Cras et al., 2002; Tang et al., 2012). VEGF therapy rescues the BPD phenotype in animal models (Kunig et al., 2005; Thebaud et al., 2005), suggesting that angiogenic factors such as VEGF may be utilized for the reciprocal crosstalk between ECs and epithelial cells in the niche at this stage. Exploration of the mechanism by which pre-natal and post-natal challenges deregulate the niche formation will lead to the development of efficient therapeutic strategies for BPD.

## Alveolar Stage (P5-30 in Mouse, 36 Weeks-8 Years Post-Natally in Human)

The final stage of lung development occurs after birth. Inner alveolar surface area significantly increases by further subdivision of the primitive alveoli through the process of secondary septation (alveolarization). After birth, dynamic environment changes such as clearance of airway fluid, expansion of the alveolar sac, increased oxygen tension and pulmonary circulation, ensue in the lung. These changes stimulate vascular growth; alveolar capillary surface area increases by ~20-fold from birth to adulthood (Zeltner et al., 1987). Lung ECs adapt to these dynamic environmental changes and regulate post-natal pulmonary circulation by secreting various vasoactive molecules including NO (Gao and Raj, 2010; Gao et al., 2016). Endothelial NO synthase (eNOS)-deficient mice exhibit paucity of alveolar capillary ECs and arrested alveolarization (Han et al., 2004), suggesting that EC-derived NO not only regulates pulmonary vascular tone, but also contributes to alveolarization at this stage.

**Figure 3:**
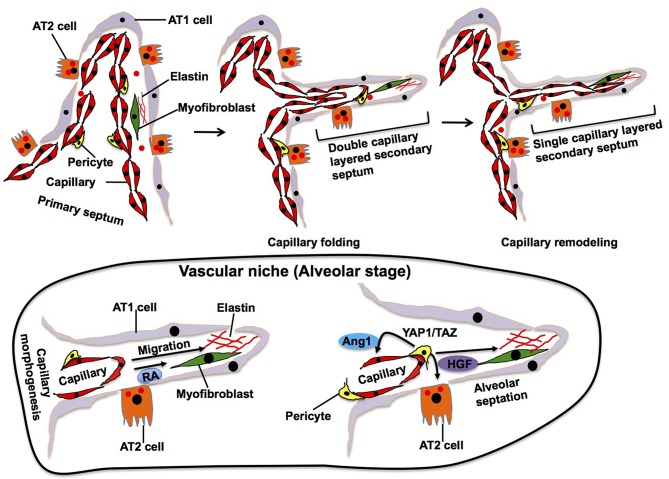
Alveolar stage. During alveolarization, alveolar area significantly increases by secondary septation, in which secondary septa subdivide the terminal sac. Elastic fibers and myofibroblasts exist at the tip of growing secondary septa. Outer layer of the capillary folds up to drive extension of the septa, in which new double-layered capillary exists (*middle*). At the later stage of alveolarization, these double layers of the capillaries in the secondary septa remodel to form a single layer of the capillary to facilitate septal thinning for efficient gas exchange (*right*). Alveolar capillary ECs produce retinoic acid (RA) and stimulate synthesis of elastin in myofibroblasts, which in turn stimulate capillary morphogenesis. Dynamic changes in the mechanical environment stimulate mechanosenstivie YAP1/TAZ signaling in pericytes to release Ang1 and HGF that act on ECs and AT2 cells and stimulate septation.

Elastin, which is synthesized and secreted by myofibroblasts, accumulates in the specific area of primary septa, and this area gives rise to a secondary crest that protrudes perpendicularly from the saccular wall into the air space forming the secondary septa (Figure 3). The outer layer of the double capillary network, existing within the primary septa, folds up to support the secondary septa in which new double-layered capillaries exist. During the extension of the secondary septa, depositions of elastin are located at the tip of the newly formed secondary septa. Elastin-derived fragments also stimulate EC migration and vascular morphogenesis (Robinet et al., 2005). Alveolar capillary ECs also produce retinoic acid (RA), which stimulate not only angiogenesis, but also synthesis of elastin in myofibroblasts (Yun et al., 2016). Although the mechanisms of secondary septation have not been well-explored, these findings suggest that reciprocal interactions between myofibroblasts and ECs in the niche drive these processes.

In later stages of alveolarization, double layers of capillaries in the secondary septa become a single capillary network via a remodeling process, called microvascular maturation, in order to create thinner septa for gas-exchange. This remodeling produces a simple and thin alveolar membrane composed of a single layer of endothelia, intermediate BM and sheet-like epithelia (AT1 cells), which facilitates efficient gas-exchange through the membrane. The mechanisms of this dynamic capillary remodeling are still unknown. However, endothelial Tie2 receptor, which plays crucial roles in vascular remodeling (Dumont et al., 1994; Suri et al., 1996; Shen et al., 2014), may play important roles in this process (Mammoto et al., 2013) because knockdown of angiopoietin (Ang)1, a ligand of the Tie2 receptor, impairs the formation of secondary septa without affecting the dynamics of elastin at the tip of the extending secondary septa (Kato et al., 2018). Pericytes, which are mesenchymal cells forming focal contacts with adjacent ECs, closely interact with alveolar capillary ECs and AT2 cells in the developing alveoli (Kato et al., 2018) and contribute to alveolarization. For example, dynamic changes of the mechanical environment in the developing lung tissues stimulate mechanosenstivie YAP1/TAZ signaling in pericytes to release Ang 1 and HGF that act on ECs and AT2 cells, respectively, during alveolarization (Kato et al., 2018). The importance of capillary ECs in the niche for alveolar development is also supported by the fact that rat alveolarization is significantly inhibited by treatment with anti-angiogenesis agents (e.g., fumagillin, thalidomide, SU5416, VEGF antagonist) (Jakkula et al., 2000; Maniscalco et al., 2002). Furthermore, loss of PECAM-1, an EC surface molecule that promotes EC migration and angiogenesis, in ECs also impairs post-natal alveolarization (DeLisser et al., 2006). Anti-PECAM-1 antibody that inhibits migration of ECs disrupts alveolar septation in neonatal rats without reducing the number of capillary ECs (DeLisser et al., 2006). Consistently, inhibition of VEGFR2 or Tie2 in ECs decreases alveolarization (Jakkula et al., 2000). These findings suggest that alveolar capillary ECs form the niche, in which ECs reciprocally interact with other resident cells to drive septal morphogenesis and capillary remodeling during alveolarization.

## Adult Lung

**Figure 4:**
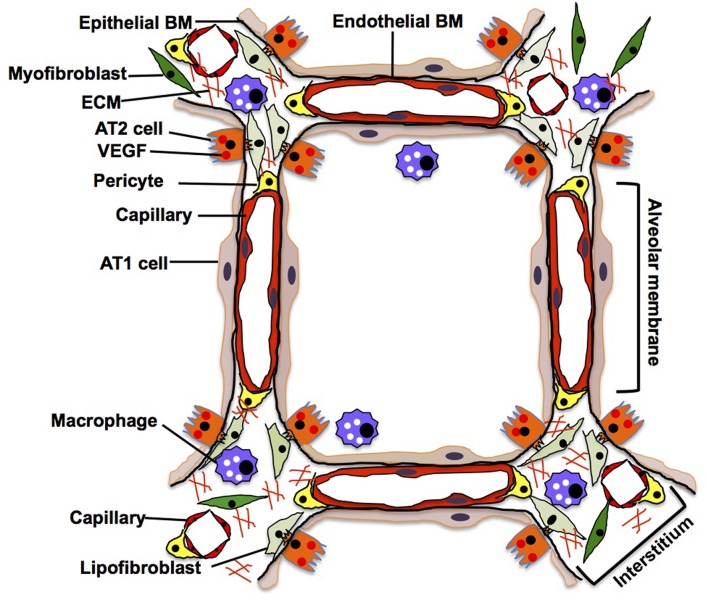
Adult lung. In adult alveoli, alveolar membrane (thin part of the alveolar wall) is simply composed of sheet-like AT1 cells, intermediate basement membrane (BM) and capillary ECs, while alveolar interstitium, a thick portion of the alveoli, consists of stromal cells (e.g., fibroblasts, pericytes, macrophages), fibrillar ECMs and separated capillaries. AT1 cells tightly share the basement membrane with capillary ECs in the alveolar membrane. In the interstitium, fibroblasts play a critical role in supporting the capillary structures by remodeling the local ECM components and supplying growth factors.

In an adult alveolus, the alveolar membrane (thin parts of the alveolar wall), through which the gas exchange occurs, is simply composed of sheet-like AT1 cells interposed by AT2 cells, intermediate BM, and capillary ECs. In contrast, the alveolar interstitium, a thick portion of the alveoli, consists of stromal cells (e.g., fibroblasts, pericytes, macrophages), fibrillar ECMs (e.g., collagens, elastin) and separated capillaries (Vaccaro and Brody, 1981; Dunsmore and Rannels, 1996) (Figure 4). Capillary ECs create a specific microenvironment with adjacent cells at both the alveolar membrane and insterstitium, and may form a distinct vascular niche similar to the niche in bone marrow (Hooper et al., 2009; Kunisaki et al., 2013; Kusumbe et al., 2014). Targeted induction of apoptosis (Kasahara et al., 2000; Tang et al., 2004; Giordano et al., 2008; Chambers et al., 2018) or senescence (Giordano et al., 2008; Kim et al., 2019) in alveolar capillary ECs leads to destruction of the alveolar structures in adult mouse. It has been demonstrated that Piezo1, a mechanosensing ion channel expressed on the lung capillary ECs, maintains homeostasis by sensing microvessel pressure and contributes to homeostasis of mouse alveoli (Friedrich et al., 2019), suggesting that alveolar capillary ECs in the niche maintain homeostasis of alveoli. By combining the use of novel site-specific EC surface markers, lineage-tracing strategies, and appropriate experimental model systems, we will be able to better understand how the specific microenvironment and function of the EC niche impacts homeostasis and regeneration of the alveoli.

AT1 cells have a thin and flat shape with multiple branches spread over a large area of the alveolar membrane; this shape facilitates their close contact with the capillary endothelium through the BM, thereby allowing efficient gas exchange (Vaccaro and Brody, 1981). Since AT1 cells share the BM with capillary ECs in the alveolar membrane (Vaccaro and Brody, 1981), alveolar capillary ECs may regulate the behaviors of alveolar epithelial cells (AT1 and AT2 cells) by supplying ECM components to the BM in alveoli (Adamson and Young, 1996; Witjas et al., 2019). Cuboidal AT2 cells that usually reside at the corners of alveoli and being a source of VEGF (Ng et al., 2001), not only produce surfactant in cooperation with interstitial lipofibroblasts (Griffin et al., 1993; Torday and Rehan, 2016), but also function as progenitor cells for AT1 cells (Evans et al., 1973, 1975; Barkauskas et al., 2013). Importantly, AT2 cells extend their cytoplasmic processes into the interstitium through the BM and closely interact with interstitial cells such as fibroblasts (Vaccaro and Brody, 1978). Although direct anatomical communication between AT2 cells and ECs has not been reported, they may communicate through the BM that can act as a sink for various growth factors [e.g., VEGF, thrombospoindin-1 (THBS1)] (Park et al., 1993; Ruhrberg et al., 2002; Tan and Lawler, 2009) as well. *In vitro* co-culture of AT2 cells and ECs demonstrated that physical contact between AT2 cells and ECs is crucial for the propagation of AT2 cells (Ding et al., 2011). Thus, there may be direct communications between AT2 cells and ECs in the alveoli. Further nanoscale imaging analysis (e.g., electron microscopy) needs to be employed to characterize the anatomical relationship between AT2 cells and ECs in the niche. Interstitial capillary ECs interact with myofibroblasts that produce lung specific ECM such as elastin and collagen to provide physical stability and elastic recoil of the alveoli (Kapanci et al., 1974). These fibroblasts support capillary structures by remodeling the local ECM components and supplying various growth factors (Hughes, 2008; Costa-Almeida et al., 2015) to maintain alveolar homeostasis (Cao et al., 2016; Mammoto et al., 2016a). Site-specific capillary ECs may constitute a distinct niche with other resident cells and maintain alveolar homeostasis.

The contribution of alveolar capillary ECs to adult alveolar regeneration has been demonstrated by the unilateral pneumonectomy (PNX) model (surgical removal of one lobe) (Leuwerke et al., 2002; Ding et al., 2011; Lin et al., 2011; Bolte et al., 2017; Mammoto T. et al., 2019). After left PNX, neo-alveolarization is induced in the remaining right lobes in adult animals (Fehrenbach et al., 2008; Ochs, 2019). Similar to developmental alveolarization, newly formed septa, which include capillaries and mesenchymal cells, arise from the preexisting septa in the alveoli of the remaining lung after PNX (Ackermann et al., 2014; Ysasi et al., 2015), suggesting that a developmental program is employed for the post-PNX alveolarization (Kho et al., 2013).

**Figure 5:**
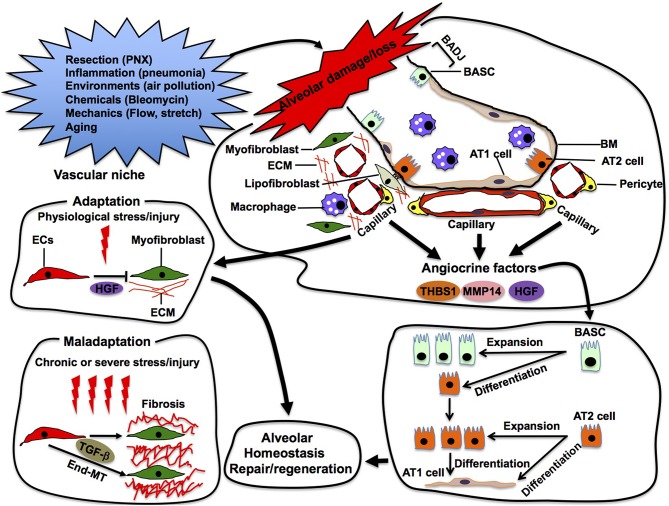
Alveolar repair in the vascular niche. Capillary ECs sense damage or loss of alveoli and produce angiocrine factors (e.g., thrombospondin 1 (THBS1), MMP14, HGF) in the vascular niche. These angiocrine factors enhance repair and/or regeneration of the damaged alveoli by stimulating propagation and/or differentiation of epithelial stem cells (e.g., AT2, BASC) in the alveoli. In the niche, ECs also adapt to physiological stress/injury to maintain tissue homeostasis. Under severe or chronic stress/injury, maladapted ECs provoke myofibroblasts, leading to pathological conditions such as fibrosis. Maladapted ECs also transdifferentiate into myofibroblasts (End-MT) to accelerate fibrosis.

Angiogenesis is one of the key events during new alveolar formation after PNX; functional alveoli cannot be formed without expansion of capillary networks. Similar to developmental alveolarization, during post-PNX alveolarization, double-layered capillaries formed by angiogenesis cover the bottom of the new alveoli and fold up inside the new septa (Ackermann et al., 2014), which is elaborately orchestrated by capillary EC signaling in the niche. It has been demonstrated that PNX activates VEGFR2 and FGFR2 on ECs to produce the angiocrine molecule MMP14 in the vascular niche. EC-derived MMP14 then cleaves the EGF-like ectodomain on EGFR to activate EGFR on the alveolar epithelial progenitor leading to the expansion of AT2 cells and bronchioalveolar stem cells (BASCs) for post-PNX alveolarization in mouse (Figure 5) (Ding et al., 2011). After PNX, the remaining lung tissues are exposed to dynamic changes in various mechanical forces (e.g., tissue distortion toward dead space, increased capillary perfusion) (Hsia et al., 2001; Dane et al., 2013, 2016), and these mechanical forces may directly or indirectly stimulate capillary ECs in the niche to accelerate alveolarization. The post-PNX lung tissues are significantly deformed in the subpleural regions (Konerding et al., 2012; Filipovic et al., 2013), and capillary ECs actively interact with AT2 cells and macrophages to expand the vascular plexus in this region (Ackermann et al., 2014). It has been demonstrated that activation of mechanosensitive YAP1 signaling in ECs stimulates angiogenesis and consequently modulates compensatory post-PNX-alveolarization through Ang/Tie2 signaling (Mammoto T. et al., 2019). Since capillary ECs are localized in the specific domains of the alveoli (interstitium and alveolar wall) that are constantly exposed to the mechanical forces (e.g., shear, stretch, deformation, perfusion pressure), ECs may act as a mechano-sensor in the niche to trigger angiogenesis and alveolarization after PNX. Further exploration of the mechano-chemical mechanism of post-PNX alveolarization is needed to fully understand the role of the vascular niche during adult lung regeneration.

In addition to physiological alveolar regeneration, capillary ECs also accelerate repair of the damaged alveoli by producing angiocrine factors (Rafii et al., 2016a). For example, bleomycin-induced alveolar injury induces expression of THBS1 in alveolar ECs, which stimulates differentiation of BASC residing at the bronchio-alveolar duct junction (BADJ) into alveolar epithelial lineages to repair damaged alveoli (Lee et al., 2014). Capillary ECs also communicate with adjacent fibroblasts and macrophages using angiocrine factors to modulate the severity of bleomycin-induced alveolar injury (Cao et al., 2016). These findings suggest that alveolar capillary ECs utilize various angiocrine signals to stimulate other cells in the niche and repair the injured alveoli in adult (Figure 5).

Although ECs adapt to physiological stress to maintain tissue homeostasis, severe and/or chronic stress leads to maladaptation of ECs in the niche. Maladapted ECs contribute to multiple pathological conditions such as fibrosis (Cao et al., 2016, 2017) and cancer (Chouaib et al., 2010; Cao et al., 2014). While EC-derived HGF prevents acute injury-induced mouse lung fibrosis by acting on perivascular fibroblasts in the niche, chronic lung injury stimulates ECs to provoke fibroblasts and induces lung fibrosis (Cao et al., 2016). Maladapted ECs in the niche also transdifferentiate into myofibroblasts (endothelial-mesenchymal transition: End-MT) through TGF-β signaling and accelerate the fibrotic lesion (Pardali et al., 2017). The maladapted ECs also activate cancer-associated fibroblasts and support cancer progression by remodeling the ECM or by secreting cytokines (Zeisberg et al., 2007). Thus, ECs may exhibit plasticity in the niche, and modulation of EC plasticity in the niche can be a promising therapeutic target for various pathological conditions such as fibrosis and cancer.

Various infectious lung diseases such as influenza and bacterial pneumonia severely damage alveoli, and the innate repair programs are employed to repair the damaged alveoli. A number of infectious lung disease models have been used to explore the mechanism of alveolar regeneration/repair in adult lung (Kumar et al., 2011; Vaughan et al., 2015; Zuo et al., 2015; Zacharias et al., 2018; LaCanna et al., 2019). However, most of these studies have focused on behaviors of the epithelium or functional aspects of the capillary ECs (e.g., vascular leakiness) (Wang et al., 2015; Birukov and Karki, 2018; Torres, 2018), and the mechanism by which ECs contribute to alveolar repair after the infectious conditions has not been well-explored. Since capillary ECs directly or indirectly modulate the local reactions to inflammation (Danese et al., 2007; Al-Soudi et al., 2017) that contribute to the alveolar damage, the role of alveolar capillary ECs in infection-induced alveolar damage and regenerative processes using the relevant animal models should be explored in the future.

It is well-known that the alveolar repair program initiates in response to environmental challenges (e.g., air pollution, smoking, viruses, bacteria). However, genetic and epigenetic status also contributes to the repair processes. Aging, which alters genetic and epigenetic conditions, may tip the balance of physiological repair process toward destruction of the alveoli. Human lung functions decline at a rate of 1% per year after around the age of 25 years even without lung diseases (Fletcher and Peto, 1977; Janssens et al., 1999; Sharma and Goodwin, 2006). Upon aging, the lung exhibits multiple age-associated changes, including increased secretion of pro-inflammatory cytokines, attenuated immune response, and alterations in structural ECM proteins (Meiners et al., 2015; Navarro and Driscoll, 2017) that are all important for the niche functions. These changes are accompanied by structural alterations such as enlarged airspaces, loss of surface area and a decrease in static elastic recoil. Importantly, it is demonstrated that one of the most significant changes in the aged lung is a decline in the number and functions of capillary ECs (Thurlbeck and Angus, 1975; Georges et al., 1978). In fact, structures of alveolar capillary blood vessels are severely disorganized with aging in mice, and ECs in the old mouse lung lose not only angiogenic ability, but also the ability to interact with alveolar epithelial cells (Mammoto A. et al., 2019). Consistently, compensatory lung growth after PNX is inhibited in the aged animals (Paxson et al., 2011; Mammoto A. et al., 2019). These findings suggest that age-associated alterations of the vascular niche may contribute to vulnerability of the aged alveoli. Given that the aging population is booming and age-associated lung diseases such as COPD and pneumonia are one of the leading causes of death in aged adults (Fry et al., 2005; Ito and Barnes, 2009; Stupka et al., 2009; Akgun et al., 2012; Lowery et al., 2013; Brandsma et al., 2017), it is critically important to investigate the effects of aging on structures and functionality of the niche to develop therapeutic options for age-associated alveolar dysfunction and diseases.

Advanced stem cell technologies, including creation of patient-derived pluripotent stem cells (iPSCs), establishment of stem cell differentiation protocols, programming of these pluripotent cells into the tissue-specific targeted cells, and targeted engraftment of the programmed cells *in vivo* have been changing the concept of current regenerative medicine. The knowledge regarding the vascular niche needs to be incorporated into the current regenerative strategy to efficiently regenerate the damaged lung. It has been demonstrated that intravenously injected ECs engraft to the mouse alveoli, accelerate post-PNX alveolar growth (Ding et al., 2011) and repair the radiation-induced organ injury (Rafii et al., 2016b). Intravenous injection of ECs from young animals also reverts the age-associated alterations of the vascular niche (decreased capillary density and losing ability to support hematopoietic stem cells) in bone marrow in old animals (Kusumbe et al., 2016; Poulos et al., 2017). Furthermore, intravenous injection of c-kit+ ECs stimulates angiogenesis and prevents alveolar simplification in the mouse BPD model (Ren et al., 2019). Although the precise mechanisms of EC engraftment need to be characterized and the efficiency of engraftment should be optimized, the approach to reconstitute ECs in the vascular niche seems to be a promising strategy to repair damaged or aged alveoli.

Organ specific acellular scaffolds that are created by treating the whole organ with a cocktail of detergents hold organ specific biomechanical cues (e.g., ECM) as well as important biochemical cues (e.g., ECM-bound growth factors) (Badylak et al., 2011; Song and Ott, 2011). These scaffolds have been utilized to engineer functional organs including lung by recellularizing the scaffolds with specific tissue-resident cells and/or iPSC-derived targeted cells (Petersen et al., 2010; Ghaedi et al., 2013; Gilpin et al., 2014; Dorrello et al., 2017). However, implanted bioengineered lung fails within several hours in animals mainly due to the inadequate maturity of the alveolar membrane (e.g., alveolar edema, hemorrhage) (Ott et al., 2010; Petersen et al., 2010). Since multiple cellular components interdependently act with ECM structures in the vascular niche and control maturation and homeostasis of the alveoli, the concept of the vascular niche needs to be leveraged to develop more efficient strategies for fabrication and recellarization of the scaffolds. More precise anatomical and functional characterization of the vascular niche in the alveoli will be necessary.

Due to the anatomical complexity of the lung tissue, there is a challenge to define the vascular niche in the lung. Various *in vitro* culture systems including organoids have been used to explore behaviors of alveolar epithelial cells and cellular interactions (Barkauskas et al., 2017). In the orthodox alveolar organoid culture, alveolar epithelial cells morphologically self-organize in the presence of stromal cells (e.g., fibroblasts) and/or differentiate into the specific lineages (Barkauskas et al., 2013; Jain et al., 2015; Frank et al., 2016). Most of the alveolar organoids exhibit a closed sphere structure (alveolosphere), which is composed of the layers of polarized alveolar epithelial cells on the BM (Barkauskas et al., 2017). Given that physiological alveoli are covered by capillary blood vessels (vascularized) to form the interfaces between the alveolar sac and ECs, modification of the conventional organoid system to further recapitulate a physiologically relevant interface will be necessary to characterize the vascular niche in the alveoli. In addition, several epithelial-endothelial co-culture studies have demonstrated that ECs stimulate patterns of lung epithelial morphogenesis (e.g., budding, branching) *in vitro* (Lee et al., 2014; Mammoto et al., 2016b; Mammoto T. et al., 2019). However, the interface between the cells including ECMs that are crucial for the formation of the vascular niche has not been visualized, and physiological mechanical forces such as blood flow and respiratory movement that are important for the EC and epithelial behaviors are not recapitulated in the systems. Modification of these systems is necessary to define the niche structure *in vitro*.

As another *in vitro* approach, the alveolar-on-a-chip system has been utilized to recapitulate the interface between alveolar epithelial cells and ECs *in vitro* for various applications (Huh et al., 2010, 2012; Benam et al., 2016; Stucki et al., 2018). Most of the systems compose of two overlapping microchannels separated by a thin, flexible, and inert microporous membrane. By culturing alveolar epithelial cells and lung microvascular ECs on each side of the membrane, either type of cells can be exposed to their respective tissue-specific microenvironment (e.g., mechanical stretch, air flow on the alveolar side and fluid flow on the vascular side). The system may be useful for exploring the effects of drugs and/or factors on behaviors of each cell type. However, since layers of each cell type are separated by an inert membrane, it is challenging to recapitulate the reciprocal morphogenetic interactions and ECM remodeling that are crucial for niche formation. Recently, a vascularized kidney organoid has been created on a chip by exposing it to laminar flow, and this organoid-on-a-chip system successfully visualizes the epithelial-endothelial interface (Homan et al., 2019). The organoid-on-a-chip system, in which alveolar epithelial cells and ECs physically interact and self-assemble into physiologically relevant alveolar units (alveolar epithelial budding covered by capillary EC plexus to form the air-blood barrier interface) in the tissue-specific mechanical environment (e.g., air and blood flow), may be able to define and explore the vascular niche in alveoli.

As a new *in vivo* approach, intravital microscopy (Looney and Bhattacharya, 2014; Yang et al., 2018) may be useful to visually characterize the vascular niche in alveoli. The lung hydrogel implantation system (Mammoto and Mammoto, 2014), in which hydrogels supplemented with the cellular and non-cellular component (e.g., growth factors, ECM) are implanted on the lung surface of a living mouse, can also be used for visualizing interactions between ECs and other lung resident cells and characterizing the specific microenvironment of the niche (Mammoto et al., 2016a, 2018; Mammoto A. et al., 2019; Mammoto T. et al., 2019). These interdisciplinary approaches may enable us to anatomically and functionally investigate the vascular niche in alveoli in the future.

## Summary

Due to exposure to an outer gaseous environment, the tissue regeneration program is constantly employed to repair the injured alveoli; deregulation of this mechanism leads to end-stage lung diseases. Therefore, it is critically important to understand the mechanism of innate alveolar repair, which will restore alveolar structures and delay disease progression. Understanding the mechanism will also help to develop cell-based regenerative therapies and/or create implantable functional lung tissues using engineering approaches such as acellular scaffolds.

During organ development and regeneration, ECs form capillary blood vessels and supply oxygen, nutrients, and cellular components to the local tissues. Besides these fundamental functions, ECs form a specific microenvironment, named vascular niche. When the niche is challenged, ECs in the niche attempt to maintain homeostasis, whereas maladapted ECs in the niche lead to various pathological conditions. Accumulating evidence that we discussed in this review indicates that capillary ECs create the vascular niche in which ECs interdependently interact with other resident cells (e.g., epithelium, fibroblasts, macrophages) and structural components (e.g., ECM) to maintain homeostasis of the alveoli. Thus, although most of the lung development and regeneration studies focus on the mechanism of alveolar epithelium repair, it is critically important to comprehensively understand the role of alveolar capillary endothelium in alveolar development, homeostasis and regeneration as well as the anatomy and functionality of the vascular niche in alveoli. Future investigation of (1) EC lineages in the niche, (2) detailed nanoscale anatomy of the niche, (3) spatio-temporal changes in microenvironment in the niche, and (4) functionalities of the distinct niche during alveolar development, homeostasis and regeneration will enable us to reverse pathological alveolar conditions (e.g., BPD, COPD) or bioengineer implantable lung tissues. These goals will be achieved by interdisciplinary approaches, which include developmental biology, imaging science, molecular and cellular biology, and engineering.

## Author Contributions

All authors listed have made a substantial, direct and intellectual contribution to the work, and approved it for publication.

### Conflict of Interest

The authors declare that the research was conducted in the absence of any commercial or financial relationships that could be construed as a potential conflict of interest.
